# Presence of early CKD-related metabolic complications predict progression of stage 3 CKD: a case-controlled study

**DOI:** 10.1186/1471-2369-15-187

**Published:** 2014-11-27

**Authors:** Herbert S Chase, Jamie S Hirsch, Sumit Mohan, Maya K Rao, Jai Radhakrishnan

**Affiliations:** Division of Nephrology, Columbia University, New York, NY USA; Department of Biomedical Informatics, Columbia University, 622 West 168th Street, New York, NY 10032 USA

## Abstract

**Background:**

Only a subset of patients who enter stage 3 chronic kidney disease (CKD) progress to stage 4. Identifying which patients entering stage 3 are most likely to progress could improve outcomes, by allowing more appropriate referrals for specialist care, and spare those unlikely to progress the adverse effects and costliness of an unnecessarily aggressive approach. We hypothesized that compared to non-progressors, patients who enter stage 3 CKD and ultimately progress have experienced greater loss of renal function, manifested by impairment of metabolic function (anemia, worsening acidosis and mineral abnormalities), than is reflected in the eGFR at entry to stage 3. The purpose of this case-controlled study was to design a prediction model for CKD progression using laboratory values reflecting metabolic status.

**Methods:**

Using data extracted from the electronic health record (EHR), two cohorts of patients in stage 3 were identified: progressors (eGFR declined >3 ml/min/1.73m^2^/year; n = 117) and non-progressors (eGFR declined <1 ml/min/1.73m^2^; n = 364). Initial laboratory values recorded a year before to a year after the time of entry to stage 3, reflecting metabolic complications (hemoglobin, bicarbonate, calcium, phosphorous, and albumin) were obtained. Average values in progressors and non-progressors were compared. Classification algorithms (Naïve Bayes and Logistic Regression) were used to develop prediction models of progression based on the initial lab data.

**Results:**

At the entry to stage 3 CKD, hemoglobin, bicarbonate, calcium, and albumin values were significantly lower and phosphate values significantly higher in progressors compared to non-progressors even though initial eGFR values were similar. The differences were sufficiently large that a prediction model of progression could be developed based on these values. Post-test probability of progression in patients classified as progressors or non-progressors were 81% (73% − 86%) and 17% (13% − 23%), respectively.

**Conclusions:**

Our studies demonstrate that patients who enter stage 3 and ultimately progress to stage 4 manifest a greater degree of metabolic complications than those who remain stable at the onset of stage 3 when eGFR values are equivalent. Lab values (hemoglobin, bicarbonate, phosphorous, calcium and albumin) are sufficiently different between the two cohorts that a reasonably accurate predictive model can be developed.

## Background

During the last decade the prevalence of chronic kidney disease (CKD) has increased considerably and is estimated to range from about 10-15% of the elderly population [1–5]. Only a portion of patients with early stage 3 CKD progress to stage 4 where the risk of cardiovascular disease, end stage renal disease (ESRD), or death becomes substantially higher [6–12]. Identifying the subset of patients who enter stage 3 and are most likely to progress to stage 4 CKD could both improve outcomes, by allowing more appropriate referrals for specialist care [13–16], as well as spare those unlikely to progress the adverse effects and costliness of an unnecessarily aggressive approach [17, 18]. This issue is particularly important in the geriatric population in whom it is unclear whether a reduced estimated glomerular filtration rate (eGFR) in the stage 3 range is associated with the poor outcomes of renal disease and in whom advancing age is associated with slower progression of kidney disease [19, 20].

While there are several useful prediction models based on various features such as the presence or absence of proteinuria, the predictive value of the majority is largely dependent on the eGFR (or creatinine) [21, 22]. Predicting the risk of progressing to stage 4 in patients newly diagnosed in stage 3, however, could only be accomplished with a model that did not use eGFR as a predictive feature given that the eGFR in all patients entering stage 3 is the same, by definition, regardless of whether the patient goes on to progress to stage 4.

We explored the hypothesis that compared to non-progressors, patients who enter stage 3 CKD and ultimately progress have experienced greater loss of renal function, manifested by impairment of metabolic function (anemia, worsening acidosis and mineral abnormalities), than is reflected in the eGFR at entry to stage 3. Presumably, progression would be less likely if the decline in GFR were due to a reduction in renal plasma flow, associated with normal aging [23–26], with minimal parenchymal loss and the absence of a distinct renal disease. The association of proteinuria and progression supports the view that when patients reach stage 3 they have greater or lesser degrees of renal injury, which influence the likelihood of progression [10, 27–29]. Proteinuria, however, is not a direct measure of functioning parenchyma but rather reflects renal injury. Not all patients with proteinuria are destined to progress, possibly reflecting a subgroup that has not suffered parenchymal loss. A more direct assessment of reduced functional parenchyma might be the presence of metabolic complications, which include reduced hemoglobin [30] and dysregulation of acid-base balance [31] and mineral metabolism [32], well-known to be present in patients with advanced chronic kidney disease. Recent investigations have demonstrated that metabolic complications can be observed as early as stage 3 and that the prevalence increases as GFR (or eGFR) declines [33–42]. Given the cross-sectional nature of these studies, however, it could not be determined if patients with early onset metabolic complications were more likely to proceed to stage 4.

The purpose of our longitudinal study was to explore the association of metabolic complications and progression of CKD. We compared average values of hemoglobin, bicarbonate, calcium, phosphorous and albumin in patients demonstrated to progress to those who did not. We also compared minimum (or maximum) values of each of these features hypothesizing that patients less able to achieve metabolic balance might experience wider swings in concentrations of metabolic lab values. To test whether any differences in metabolic lab data observed between progressors and non-progressors were of sufficient magnitude to serve as useful attributes in a prediction model, we used classification algorithms to create a model based exclusively on these lab data.

## Methods

### Identification of patients with progressive or non-progressive stage 3 CKD

The data source for this study was the clinical data warehouse of the Columbia University Medical Center (CUMC) of the New York Presbyterian Hospital. Patients whose records were used in this study were regularly cared for in the Associates of Internal Medicine (AIM) primary care clinic for adults (age 21 and older) on the CUMC campus. The study was performed with the approval of the CUMC Institutional Review Board in compliance with the Helsinki Declaration. The Institutional Review Board did not require written informed consent for participation in the study and granted a waiver given the nature of the study (to analyze data from the data warehouse, which is not considered a potential risk to a patient).

**Figure 1:**
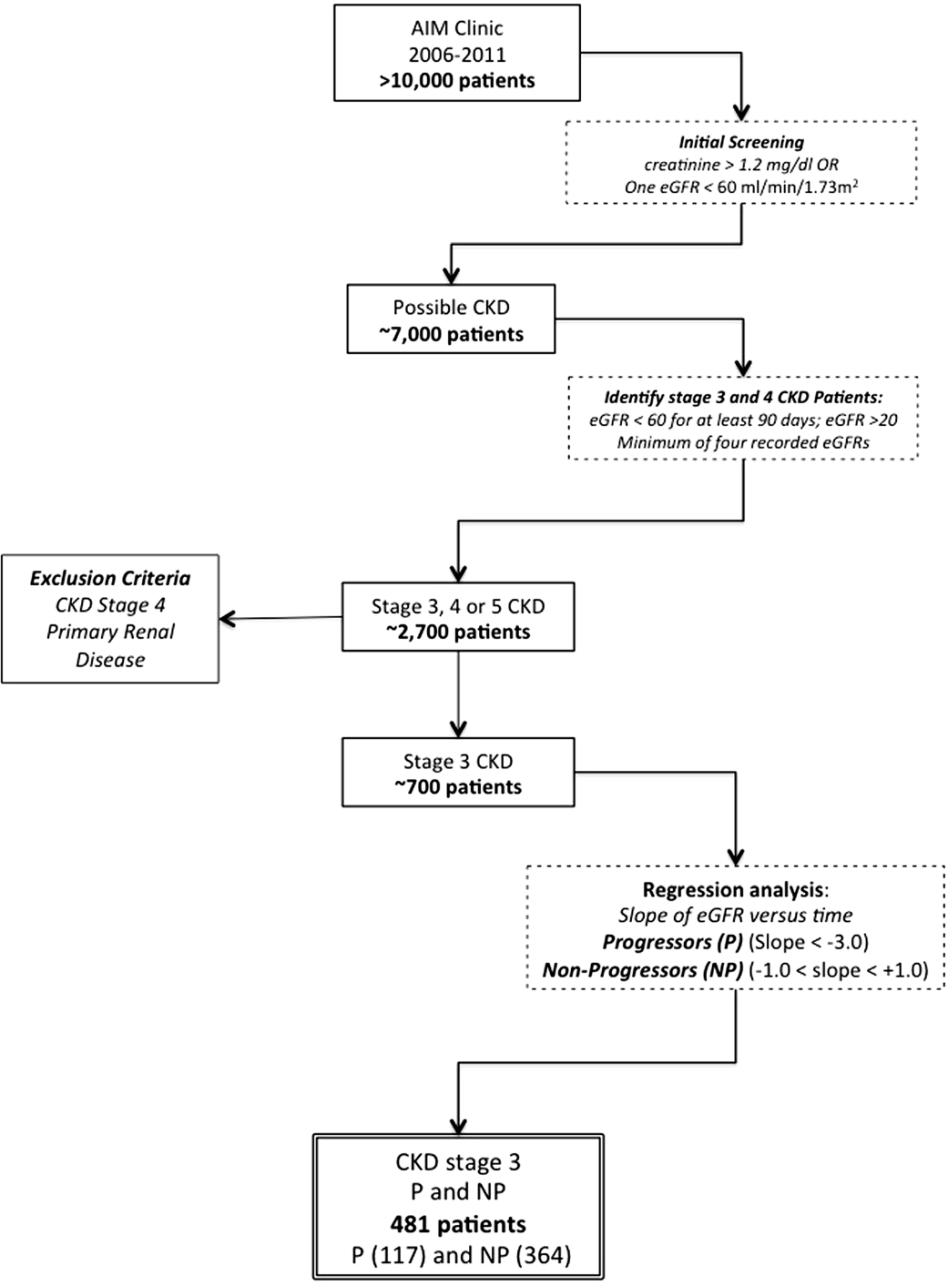
**Patient selection algorithm.**

Our goal was to identify a cohort of patients who both met the current National Kidney Foundation definition of stage 3 CKD (KDOQI, [13]) and had sufficient data in their record to determine whether or not their eGFR had either progressively declined or remained stable over several years. From an initial screen of over 10,000 patients cared for in the AIM clinic, from 2006-2012, a cohort of approximately 700 were identified with stage 3 CKD (patient selection is summarized in Figure 1). Estimated GFR for each patient was calculated using the four-variable Modification of Diet in Renal Disease (MDRD) formula [43] based on the patient’s creatinine values, gender, race (African American or not), and age, data extracted from the clinical data warehouse. Standardization of creatinine measurements is calibrated to IDMS. Only patients with 5 visits or more were included to ensure sufficient data to determine if they were progressors of non-progressors. Patients were excluded if they were consistently in stage 4, had received a kidney transplant, had ICD-9 code documentation of diseases known to cause rapid loss of renal function (primary glomerulonephritis, HIV-AIDS, or systemic lupus erythematosus) or of nephrotic syndrome. Patients were followed an average of 6.0 (SD 2.9) years and the majority (87%) had four consecutive years of data available.

Progressors (P) and non-progressors (NP) were identified on the basis of the slope of the changes of eGFR over time [44]. For each patient with at least 4 or more measurements of creatinine over a period of four years, a linear regression of eGFR versus time was performed yielding a slope and standard error. Progressors were defined as those patients whose eGFR declined at a rate greater than 3 ml/min/1.73m^2^ per year (slope of < −3.0 ml/min/1.73m^2^/year), significantly different from 0 (*p* <0.05) [45, 46]. Non-progressors were defined as those patients whose eGFR did not decline meaningfully over time (slopes between −1.0 and +1.0 ml/min/1.73m^2^/year). There were 117 and 364 P and NP patients, respectively.

### Data acquisition

Lab data and diagnosis data for co-morbid conditions were extracted for analysis and classification. Labs extracted included hemoglobin, bicarbonate, phosphorous, calcium, albumin and dipstick protein in a urinalysis. For analysis and model-building the dipstick results were converted to numbers: none = 0; trace = 0.5; 1+, 2+, 3+, 4+ = 1, 2, 3 and 4, respectively. Diagnosis data was recorded in the EHR using International Classification of Diseases, version 9 (ICD-9) codes. Two data sets were generated: *initial* and *follow-up*. Initial data was collected for each patient from one year before the start date of sustained stage 3 CKD through one year after. Follow-up data on these same patients was collected when they had been in stage 3 (or higher) for more than 3-5 years (average of approximately 5 years).

### Predictive modeling

Using the Weka workbench [47], we developed a predictive model using logistic regression and Naïve Bayes on a *training set* (the follow-up data) when patients had been in stage 3 for several years. The model was then used on the *testing set* (the initial data) to see how well the model could predict which patients were likely to progress when they first entered stage 3. Models were validated and accuracy assessed by calculating sensitivity, specificity, and the area under the curve (AUC) of the receiver-operating characteristic (ROC). Patients were also randomized into separate training and testing sets; models built using half the patients were tested on the lab values of the other half of the patients.

### Statistics

For continuous values, the Student t-test was used to determine significance. For categorical values, the Chi-squared test was used. Regression analysis was conducted using standard methodology implemented through a Java framework. Likelihood ratios and positive and negative predictive values were calculated from sensitivity and specificity. Post-test probabilities were calculated from likelihood ratios using logarithmic transformation [48].

## Results

### General characteristics of progressors and non-progressors

P were significantly younger than NP: average ages were 71.3(SD 11) and 75.9 (SD 11), respectively (Table 1). The percentage of progressors who were African American was significantly higher (7.6%) than in non-progressors (2.5%) (*p* <0.01). There was no significant gender difference between the two groups: 61.3% of progressors were female compared to 65.2% of the non-progressors (*p* = 0.59). Though a sizable proportion of both P and NP had diabetes ICD-9 codes in their EHR (75.6%, 62.8%, respectively); the proportion was significantly higher in P (*p* = 0.015). CKD progression was not significantly associated with having received an ICD-9 code for hypertension.

**Table 1:** **Demographics and co-morbid conditions of NP and P patients**

Patient	Attribute	NP	P
characteristics			
Demographics	Female	65.2%	61.3%
	African American**	2.5%	7.6%
	Age**	75.9 (SD 11)	71.3 (SD 11.7)
Co-Morbid conditions	Diabetes*	62.8%	75.6%
	Hypertension	99.2%	100%

Significance levels: **p < 0.01; *p <0.05.

### Variability of lab values

There was significant intra-patient variation of eGFR and other laboratory values. Fluctuations of eGFR in patients who did not progress over many years showed swings as wide as 40 ml/min/1.73m^2^. Given the intra-patient variability, we calculated and compared average initial values over a 2 year time period, a year before and a year after the start date of stage 3 CKD (see below) for each patient to be used in the analysis and modeling. We also extracted the minimum and maximum values. The coefficients of variation and the difference between the average maximum and minimum values of several laboratory tests are presented in Table 2.

**Table 2:** **Intra-patient variation of lab values**

Attribute	Coefficient of variation	Maximum-minimum spread
eGFR	0.14 (SD 0.11)	12 (SD 9), ml/min/1.73m^2^
Phosphorous	0.13 (SD 0.13)	1.8 (SD 1.6), mg/dl
Bicarbonate	0.09 (SD 0.05)	6.8 (SD 4.5), meq/l
Potassium	0.09 (SD (0.01)	1.3 (SD 1.3), meq/l
Hemoglobin	0.06 (SD 0.06)	2.2 (SD 2.2), g/dl

### Identification of CKD stage 3 start date

While most patients who entered stage 3 CKD remained in stage 3 (or higher) for the duration of their electronic record of data, there were some whose eGFR intermittently rose above 60 ml/min/1.73m^2^, an observation expected following the demonstration of the large coefficient of variation of eGFR values (Table 2). We chose the date on which patients developed sustained stage 3 CKD to be that when eGFR remained consistently below 60 ml/min/1.73m^2^ for at least one year.

### Lab values reflecting metabolic complications of renal disease

Progressors and non-progressors had similar initial average eGFR values at the time of entry to stage 3 (Table 3). Progressors, however, were found to have significantly lower *minimum* values of eGFR at the time they entered stage 3. Average values of both P and NP were below the usual stage 3 cut-off of 59 ml/min/1.73m^2^ due to periodic dips of eGFR to values below 40 ml/min/1.73m^2^ (see MIN, Table 3) and reflecting the requirement that patients’ eGFR was <60 ml/min/1.73m^2^ for at least one year rather than the KDOQI defined 90 days. Average and minimum (or maximum) initial values for plasma bicarbonate, hemoglobin, calcium, phosphorous, and albumin were significantly different between P and NP.

**Table 3:** **Lab tests values**

Lab value		***Class***	***Initial (Testing)***	SIG	***Follow-up (Training)***	***SIG***
eGFR *[100%]*	MIN	P	34		20	
		NP	39		34	
		Δ	*−5*	***	*−14*	***
	MAX	P	57		44	
		NP	58		49	
		Δ	*−1*	NS	*−5*	***
	AVG	P	47		34	
		NP	48		44	
		Δ	*−1*	NS	*−10*	***
HEMOGLOBIN ***[92%]***	MIN	P	10.9		9.6	
		NP	11.5		11.0	
		Δ	*−0.7*	**	*−1.4*	***
	AVG	P	12.0		11.3	
		NP	12.6		12.3	
		Δ	*−0.5*	***	*−1.0*	***
BICARBONATE *[100%]*	MIN	P	20.7		16.9	
		NP	21.8		19.6	
		Δ	*−1.1*	**	*−2.7*	***
	AVG	P	24.1		23.0	
		NP	25.1		24.5	
		Δ	*−0.9*	***	*−1.4*	***
CALCIUM *[100%]*	MIN	P	8.5		7.9	
		NP	8.6		8.2	
		Δ	*−0.2*	*	*−0.4*	***
	AVG	P	9.1		9.0	
		NP	9.2		9.2	
		Δ	*−0.1*	***	*−0.2*	***
PHOSPHOROUS *[53%]*	MAX	P	4.1		5.2	
		NP	3.7		4.3	
		Δ	*0.4*	**	*0.9*	***
	AVG	P	3.6		3.9	
		NP	3.3		3.4	
		Δ	*0.3*	***	*0.4*	***
ALBUMIN *[95%]*	MIN	P	3.7		3.1	
		NP	3.9		3.5	
		Δ	*−0.2*	**	*−0.5*	***
	AVG	P	4.0		3.9	
		NP	4.1		4.1	
		Δ	*−0.2*	***	*−0.2*	***
DIPSTICK PROTEIN ***[57%]***	MAX	P	1.4		2.2	
		NP	0.5		0.9	
		Δ	*0.9*	**	*1.4*	***
	AVG	P	1.1		1.4	
		NP	0.4		0.5	
		Δ	*0.7*	***	*1.0*	***

Displayed are average, minimum or maximum values of P and NP, and the difference between them (Δ). Initial and follow-up data (see Methods) are presented separately**.** Negative numbers indicate that the value is lower in P; positive numbers, higher. Statistical significance of differences: ***p <0.001; **p <0.01; *p <0.05; NS, not significant. % of values under test name indicate proportion of patients having had the test.

Follow-up data, used as the training set for classification model building, are also presented in Table 3. As expected, average and minimum eGFR values collected from patients who had been in stage 3 or 4 for several years were significantly lower in P versus NP. The differences in hemoglobin, bicarbonate, calcium and phosphorous between P and NP, observed in the initial data, was magnified in the follow-up data. Parathyroid hormone levels were significantly higher in P than NP, 179 (SD 132) and 95 (SD 86) pg/ml (*p* <0.01), respectively. 1, 25 Vitamin D levels were significantly lower in P than NP, 26 (SD 11) and 38 (SD 15) ng/ml (*p* <0.005), respectively. However, too few patients had either PTH or Vitamin 1,25 D values measured during the initial period, at the time of entry to stage 3, to provide for a meaningful comparison between P and NP.

### Proteinuria

Fewer than 60% of patients had results of a urinary dipstick for protein in their electronic health record. Of these patients, the initial average and maximum values of qualitative urine protein were significantly different between P and NP (Table 3). Approximately 50% of progressors had significant proteinuria (2+ - 4+) while 70% of non-progressors had little or no proteinuria (0-trace).

### Predictive modeling using lab data

To determine if the differences in initial lab data (Table 3) between the progressors and non-progressors at the time of entry to stage 3 was predictive of progression, we used classification algorithms, generated from the training set, on the initial data (testing set). The eGFR data was not included as an attribute for model building given that the initial values were similar in P and NP. Sensitivity, specificity and positive and negative likelihood ratios generated by the classification are presented in Table 4. Naïve Bayes generated the higher sensitivity, 72% (CI 63% −80%) and logistic regression generated the higher specificity, 89% (CI 85% −92%). The ROC AUC was similar for both algorithms (0.73, 0.75, respectively). Post-test probabilities of either being a progressor or non-progressor, based on classification, were calculated assuming a prevalence of progression of approximately 40% [11]. Using the higher specificity generated by logistic regression and the higher sensitivity generated by Naïve Bayes yielded post-test probabilities for classification as a progressor or non-progressor were 81% (73% − 86%) and 17% (13% − 23%), respectively.

**Table 4:** **Accuracy of classification algorithms based on initial metabolic data laboratory values**

	Naïve Bayes		Logistic regression	
***Statistic***	***Value***	***95% confidence***	***Value***	***95% confidence***
Sensitivity	72%	63% −80%	43%	34% −52%
Specificity	65%	60% −70%	89%	85% −92%
LR+	2.06	1.72 − 2.46	3.79	2.66 − 5.42
LR-	0.43	0.32 − 0.58	0.65	0.55 − 0.76
PPV	58%	53% −62%	72%	64% −78%
NPV	22%	18% −28%	30%	27% −34%

## Discussion

### CKD-related metabolic complications in patients who progress

The purpose of this study was to design a prediction model for CKD progression using laboratory values other than eGFR based on the hypothesis that patients who enter stage 3 and ultimately progress have sustained a greater loss of renal function, manifested by impairment of metabolic function, than is reflected in the eGFR at stage 3 entry, compared to non-progressors. Future progressors had already developed metabolic complications by the time they entered stage 3 CKD, despite no differences in initial eGFR between progressors and non-progressors. Hemoglobin, bicarbonate, calcium, and albumin levels were significantly lower, and phosphate significantly higher in the progressors (Table 3). The differences between the two groups were magnified when maximum or minimum values were compared. These results confirm and extend prior work demonstrating that metabolic complications can be observed in patients as early as stage 3 and that the prevalence increases as GFR (or eGFR) declines [33–41]. Our study, which follows patients longitudinally, directly demonstrates the association of metabolic complications and progression. In our study, given that these laboratory measurements are measured on most patients in a screening metabolic panel far more than urine protein measurements, the ability to use them in a prediction model for CKD progression could be very useful.

It is unclear why future CKD progression is associated with early metabolic complications. One explanation is that future progressors have an ongoing disease process, which results in greater parenchymal injury and metabolic complications, which is absent in non-progressors, and not yet reflected in the eGFR. One example of an ongoing disease process inflicting damage would be diabetes. In our cohort, there was a significantly higher prevalence of diabetes in progressors than in non-progressors (Table 1). This difference, however, was small, possibly due to sample size not being powered to look for this difference. Furthermore, given the use of ICD-9 codes, we could not distinguish between a patient with diabetes and concomitant and unrelated CKD, and a patient felt to have CKD from diabetes. On average, progressors had a higher level of proteinuria (Table 3), possibly reflecting a higher prevalence of diabetic kidney disease in this group. Lastly, we did not assess either the degree of glycemic control [49] or the use of RAS inhibitors to control hypertension. If the use of RAS inhibitors was higher in the patients with diabetes who did not progress, this difference could explain why some patients with diabetes progressed and others did not. Given our exclusion criteria, it is also unlikely that the progressors suffered from a primary disease, such as glomerulonephritis, known to cause progressive renal failure. It thus remains unclear what underlying process might be responsible for early metabolic abnormalities being associated with future CKD progression but seems likely that it represents an early loss of non-filtration function that can be used to categorize patients into higher and lower risk groups.

Our observation that non-progressors do not manifest metabolic complications suggests that these patients might have less parenchymal loss than the progressors. The basis of the reduced eGFR may be due to diminished blood flow with reduced renal reserve preventing compensatory hyperfiltration [50–52]. Not distinguishing between these two groups and using the term “chronic kidney disease” to characterize patients in whom there is only a decline in the glomerular filtration without other manifestations of disease is not clinically useful [18, 53, 54]. A better term for such patients might be “chronic renal insufficiency” (CRI) a previously commonly used term that that was retired with the introduction of the KDOQI staging guidelines [18, 54]. The distinction between CKD and CRI is particularly important in the elderly given the declines in eGFR related to aging and reduced renal reserve [12, 23, 52]. Lower eGFRs may inappropriately label subjects with CKD when in fact they have only age related declines in renal function and are unlikely to have progressive renal disease. Compensatory hyperfiltration at the early stages of reduced renal mass only adds to the difficulty associated with identifying patients with true renal disease. In addition to possibly leading to over-referral and treatment of patients without true kidney disease, this lumping of all patients with reduced eGFR into the same stages also leads to an inability to target those who are most at risk to progress at an early stage when perhaps intervention has the most hope of improving outcomes.

### Current staging of CKD

The apparent uncoupling of filtration (eGFR) and metabolic renal function suggests that the current staging paradigm, based primarily on eGFR for stages 3 and higher, is not useful in estimating the degree of renal parenchymal injury and likelihood of progression. More recent modifications of staging subdivide stage 3 into stage 3A (<60 and >45 ml/min/1.73m^2^) and stage 3B (<45 and >30 ml/min/1.73m^2^) and take into consideration the degree of proteinuria. [55]. While the presence of proteinuria clearly signifies renal injury, and is predictive of progression [10, 28], it does not directly reflect loss of non-filtration renal function. Nearly half our patients who progressed and manifested complications of CKD did not have significant proteinuria. Furthermore, of our own cohort of patients with established CKD, routinely seen in clinic for an average of 5 years, nearly half did not have a urine dipstick measurement, making it more difficult to use proteinuria as a marker of progression in real clinical practice. It has been suggested that the staging algorithm consider the presence or absence of metabolic renal complications [38] which are currently not included [56]. Following the KDOQI guidelines to screen for metabolic complications upon entry to stage 3 [13] would enable physicians to assess the degree to which they are present.

### Inter-patient variability of eGFR values

Our study showed a substantial inter-patient fluctuation of eGFR likely the results of the inherent variability of the creatinine measurement [57] and changes in volume status, blood pressure, or the effect of medicines taken. The variability poses several problems for classifying patients and predicting outcomes. First, patients diagnosed with stage 3 CKD using the KDOQI guideline of 90 days might subsequently be found to be in stage 2 when their eGFRs rise above 60 ml/min/1.73m^2^ [56]. Prior studies have found that over 20% of patients identified as being in stage 3 move back into stage 2 sometime later [58]. For these reasons, we defined stage 3 as of eGFR <60 for at least one-year. Second, recently developed tools that provide a prediction of the time course to end stage renal failure [22, 31] if based on a single value, would provide widely disparate estimates of the probability of progression. However, if the algorithm were based on average values rather then individual ones, the accuracy of the staging would be improved and more consistent. Third, wide eGFR fluctuations will complicate the use of decision support tools designed to identify CKD patients through electronic health records. Studies have shown poor detection of CKD [59–62] suggesting the potential utility of automated method to identify patients with CKD through the electronic health record. If the patients are coded stage 3 only to have that reversed on subsequent measurements of eGFR, providers will lose confidence and ignore the notifications.

### Predicting which stage 3 patients are likely to progress

The sooner it can be established that a patient is likely to progress from stage 3 to stage 4 CKD, the earlier referral and aggressive intervention could be implemented [13–16]. Accurate prediction might also reduce over-referral, which subjects patients to unnecessary treatment and results in over utilization of limited resources.

To calculate the risk of CKD progression, investigators have focused on conditions that cause renal decline (such as diabetes or hypertension), abnormalities that result from renal injury (such as proteinuria) [10, 17, 21, 39, 63] or factors known to accelerate progression (such as smoking or obesity) [9, 64, 65]. Some models use end stage renal disease or death as an endpoint [30, 31, 66] while others use progression itself [45, 46, 67, 68].

The majority of currently available prediction models are based primarily on the eGFR or creatinine; the lower the eGFR the greater the risk of progression to end stage [21, 22]. The use of serum creatinine to predict CKD progression is particularly challenging in elderly patients in whom changes in muscle mass make creatinine a less accurate marker for GFR. Models that attempt to distinguish individuals with age related decline in renal function from those with truly impaired renal function, and at risk of continued progression as a result, might be more accurate in using other measures of renal parenchymal function besides eGFR.

Our observation that metabolic complications occur early in stage 3, are associated with progression, and have predictive value suggests that models should include markers of metabolic status laboratory data in the model. This is especially true if metabolic complications, once developed, also accelerate progression [34, 44, 52, 69–73]. In our model based solely on metabolic lab data, post-test probabilities for progression or non-progression were 81% (73% − 86%) and 17% (13% − 23%), respectively, sufficiently accurate to serve as a useful clinical tool. The ROC AUC achieved by the classifier (~0.74) compares favorably to those models that rely on eGFR or creatinine [22]. Halbesma et al developed a renal risk score to predict progression of CKD and demonstrated superior predictive accuracy (ROC AUC was 0.84) [45] but included the initial eGFR value as a feature and which contributed the most to the renal risk score. Our own classification statistics should improve as more patients have key metabolic lab values (PTH, vitamin D) recorded in the EHR, known to be abnormal early in the course of renal disease [74, 75].

### Limitations

There are several limitations to our study. First, the total number of patients in the P and NP group was less than 500. It is important to confirm the differences in metabolic lab values between progressors and non-progressors and to validate the classification models with a larger set of patients. Second, it is possible that observations of early metabolic abnormalities in stage 3 patients from the CUMC AIM clinic patients, consisting of primarily older, female, non-white patients (Table 1) might not be observed in younger, male and white patients. Future studies will be required to determine if the same differences are seen in a different demographic. Third, we used the MDRD formula to calculate eGFR rather than the CKD-EPI formula. Recent studies have demonstrated the MDRD calculation tends to underestimate the eGFR [76, 77]. However, the average MDRD eGFR values in both cohorts were significantly below 60 ml/min/1.73m2, suggesting that patients were in stage 3 regardless of the method used to calculate eGFR. Also, the two cohorts (P and NP) were chosen on the basis of the slope of the eGFR versus time, which is largely independent of the method of calculation. Our conclusions regarding the association of presence of metabolic complications and progression are thus unaffected by the method chosen to calculate eGFR. Fourth, although we eliminated patients with primary renal disease from the cohorts based on their ICD-9 codes, the known inaccuracy of ICD-9 coding [78] cannot eliminate the possibility that some patients who progressed actually had a primary renal disease. Last, we used a linear regression model to classify individuals into progressors vs. non-progressors, an approach that does not account for the intra-individual correlations of repeated GFR estimates over time. This approach might have resulted in misclassification of some of the patients. This limitation will be addressed in future studies by using a mixed effects model to classify patients.

## Conclusions

Our studies demonstrate that patients who enter stage 3 and ultimately progress to stage 4 manifest a greater degree of metabolic complications than those who remain stable at the onset of stage 3 when eGFR values are equivalent. Lab values (hemoglobin, bicarbonate, phosphorous, calcium and albumin) are sufficiently different between the two cohorts that a reasonably accurate predictive model can be developed.
